# Disseminated Histoplasmosis in a Patient With Acquired Immunodeficiency Syndrome: A Case Report and Literature Review

**DOI:** 10.7759/cureus.109985

**Published:** 2026-05-31

**Authors:** Aishwarya Saripalli, Colby Kulyn, Jigar Patel, Shikha Mishra, Rasha Kuran

**Affiliations:** 1 Internal Medicine, Kern Medical Center, Bakersfield, USA; 2 Internal Medicine/Infectious Diseases, Kern Medical Center, Bakersfield, USA

**Keywords:** diffuse histoplasmosis, hiv aids, invasive fungal infections, non-endemic histoplasma, treatment guidelines

## Abstract

Histoplasmosis is an endemic mycosis in the central and eastern USA, Central and South America, parts of Asia, and Africa. It commonly presents as a pulmonary disease, but can involve multiple organ systems in immunocompromised individuals. In patients with HIV and low CD4 counts, usually <150 cells/μL, it can present with gastrointestinal symptoms, lymphadenopathy, and rarely as meningitis, or with mucocutaneous and adrenal involvement. Diagnostic modalities include direct visualisation of the fungus in tissue specimens, Histoplasma antigen detection in blood or urine, antibody detection, detection of DNA sequences by PCR, and culture of clinical specimens. Treatment is based on disease severity, with a long course of itraconazole and induction therapy with amphotericin B for moderate to severe infection. HIV patients with a CD4 count of <150 cells/μL in endemic areas with high disease incidence require prophylaxis. We report a case of a 35-year-old male with HIV infection who presented with diarrhoea and abdominal pain. Evaluation revealed retroperitoneal, mesenteric, supraclavicular lymphadenopathy, splenomegaly, ileitis, and colitis. Terminal ileum mucosa and supraclavicular lymph node biopsy specimens showed *Histoplasma capsulatum* on pathology with a positive tissue fungal culture. He was successfully treated with Itraconazole with symptomatic improvement.

## Introduction

Histoplasmosis is a prevalent endemic fungal infection among patients with acquired immunodeficiency syndrome (AIDS) [1]. In this population, the disease more often presents in a disseminated form, whereas in immunocompetent individuals living in endemic regions, it typically presents with localised pulmonary involvement [1]. In patients with HIV and low CD4 counts, usually <150 cells/μL, it can present with gastrointestinal symptoms, lymphadenopathy, or mucocutaneous and adrenal involvement and rarely as meningitis [1]. Cases of histoplasmosis in non-endemic regions are usually reactivation of latent infection, remote exposure, which was asymptomatic or unrecognized exposure. We present a case of disseminated histoplasmosis in a patient with AIDS in a non-endemic region with no reported travel history, which makes recognizing this disease as a differential diagnosis challenging. 

## Case presentation

**Figure 1:**
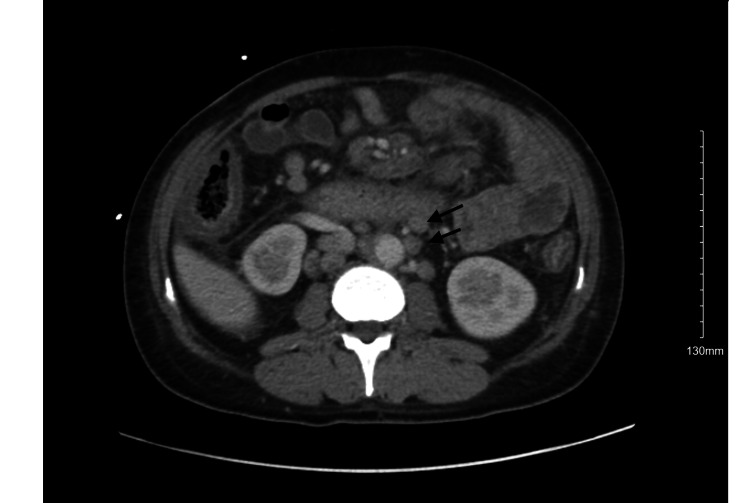
Left para-aortic and retroperitoneal lymphadenopathy (shown by the arrows)

A 35-year-old male with HIV, diagnosed in 2017, not compliant with treatment, with treated late latent syphilis and methamphetamine use, presented to the emergency room with diffuse abdominal pain and watery diarrhoea over the past month. He lived in California all his life and reported no travel to any endemic regions. His vital signs were remarkable for tachycardia, and he was otherwise normotensive and afebrile. Physical examination revealed diffuse abdominal tenderness, bilateral pedal edema, and oral thrush. Prior history included admission for dysphagia three months prior due to candidiasis, and he was treated with fluconazole with subsequent improvement in dysphagia. Laboratory results on admission were noteworthy for anemia with hemoglobin of 6.5 g/dL, HIV RNA quantitative PCR 150,000 copies/mL, and absolute CD4 count <20 cells/microliter (Table 1). CT abdomen and pelvis done on admission showed mesenteric and retroperitoneal lymphadenopathy (Figure 1), colitis, distal ileitis, and splenomegaly. Evaluation for the etiology of his diarrhoea, including stool ova and parasite testing, stool cultures, stool *Cryptosporidium* antigen, stool *Isospora* and *Cyclospora* examination, stool *Giardia* antigen, *Clostridioides difficile* testing, gastrointestinal polymerase chain reaction (PCR) panel (which included the* Campylobacter* group, *Salmonella* species, *Shigella* species, Shiga toxin, *Vibrio* group, *Yersinia enterocolitica*, rotavirus, and norovirus) were all negative. Colonoscopy with biopsy was done on day 3 of hospitalization, which showed multiple ulcerations and mucosal erythema in the terminal ileum, ileocecal valve, cecum, ascending colon, and moderate patchy mucosal erythema in the descending colon. Pathology results of the terminal ileum biopsy specimen showed GMS stain positive for fungal organisms, which were confirmed to be *Histoplasma*. *Histoplasma* antigen measured in blood and urine was positive, above the level measurable by the test. Since the patient was clinically stable, without evidence of end-organ damage, he was treated as moderate disseminated histoplasmosis with azole therapy. He was initiated on itraconazole therapy (200 mg three times a day for three days, followed by 200 mg two times a day). He was simultaneously started on anti-retroviral therapy (dolutegravir/abacavir/lamivudine; noted to have AZT (zidovudine) resistance) and prophylaxis for *Pneumocystis jiroveci *due to low risk for paradoxical immune reconstitution inflammatory syndrome with histoplasmosis. In addition, an enlarged left supraclavicular lymph node biopsy was performed, positive for *Histoplasma capsulatum *on pathology. Fungal culture of the lymph node biopsy tissue was also positive for *Histoplasma* at day 10. Blood cultures for bacteria and fungal organisms were negative. He was discharged with outpatient infectious disease follow-up for continued management of HIV and response to treatment for disseminated histoplasmosis and appropriate therapeutic drug monitoring. He was subsequently seen in the infectious disease clinic four weeks later and reported improvement in diarrhoea with Itraconazole treatment.

**Table 1:** Laboratory results HIV RNA PCR: human immunodeficiency virus ribonucleic acid polymerase chain reaction

Test name	Result	Reference range
Hemoglobin	6.5 g/dL	13.2-17.4 g/dL
HIV RNA PCR quantitative	150,000 copies/mL	Not detected
Absolute CD4 lymphocyte count	<20 cells/microL	490-1740 cells/microL

## Discussion

Histoplasmosis, the most prevalent endemic fungal infection in the United States, is caused by the environmental fungus *Histoplasma capsulatum*. Two distinct variants exist: *Histoplasma capsulatum var. capsulatum *and* Histoplasma capsulatum var. duboisii*, the latter being geographically restricted to Africa [1].

Disseminated histoplasmosis is an AIDS-defining illness and occurs in approximately 5% of patients with AIDS residing in endemic regions of the United States, particularly the Mississippi and Ohio River valleys [2]. In HIV-infected individuals, it is typically associated with advanced immunosuppression, particularly when CD4+ lymphocyte counts fall below 150/μL [1]. Immunity against *H. capsulatum* primarily relies on T cells and activated macrophages [3]. Disseminated disease arises as a result of a new infection and occasionally from the reactivation of a latent infection. Most cases in immunocompetent individuals remain asymptomatic; however, exposure to a high fungal inoculum can lead to severe pulmonary disease even in healthy hosts [1].

Pulmonary histoplasmosis can range from acute pulmonary histoplasmosis to chronic cavitary histoplasmosis [1]. Acute pulmonary histoplasmosis resembles bacterial community-acquired pneumonia, with similar symptoms and focal airspace disease on imaging, but is often accompanied by hilar or mediastinal lymphadenopathy. Severe cases may progress to acute respiratory distress syndrome and hypoxic respiratory failure [1]. Chronic cavitary histoplasmosis tends to affect older adults with underlying pulmonary disease and clinically mimics reactivated tuberculosis. Cavitation occurs in about 39% of chronic cases [4].

In immunocompromised hosts, even minimal exposure can lead to severe disseminated disease. Disseminated histoplasmosis can involve nearly any organ system, and autopsy studies frequently reveal subclinical involvement [5]. Gastrointestinal manifestations include abdominal pain (35%) with or without diarrhea (35%), while ascites, lower GI bleeding, and obstruction are rare (<5%). Hepatomegaly (28%) with or without splenomegaly (16%) may be noted on examination [6,7]. Gastrointestinal tract involvement is often clinically silent but may result in ulcerations, polypoid lesions, or perforations, particularly in the terminal ileum and colon, occurring in up to 70% of cases [5]. Lymphadenopathy is present in 65% of cases, with superficial nodes (cervical, axillary, and inguinal) affected more commonly than deep nodes (hilar, mediastinal, and intra-abdominal) (48% vs. 40%) [7]. Meningitis develops in fewer than 10% of disseminated cases and carries high morbidity and mortality [7]. Diagnosis is often delayed, particularly in immunocompetent individuals presenting with chronic meningitis [8]. Bone marrow involvement is frequent, contributing to cytopenias such as anemia (89%), neutropenia (40%), and thrombocytopenia (37%), with pancytopenia occasionally seen [7]. Less frequent findings include mucocutaneous lesions (1-7%), adrenal insufficiency, and secondary hemophagocytic lymphohistiocytosis (HLH) [1,6].

A variety of diagnostic tools are used for histoplasmosis, with performance varying by disease manifestation and host immune status due to differing fungal burdens [9]. Diagnosis can be made or supported based on clinical presentation, culture, histopathology, antigen and/or antibody testing, or polymerase chain reaction (PCR) [10]. Culture remains the diagnostic gold standard and can be performed on specimens, such as sputum, blood, bone marrow, or affected tissues. Bone marrow and blood cultures offer about 75% sensitivity for disseminated disease [11]. Overall, culture sensitivity ranges between 50% and 85%, depending on the disease type [12]. Direct microscopy can aid diagnosis when positive. Antigen detection in blood, urine, cerebrospinal fluid (CSF), and bronchoalveolar lavage (BAL) is useful, especially for disseminated or acute pulmonary disease in advanced HIV infection [11,13]. However, sensitivity is lower for chronic pulmonary histoplasmosis due to the lower availability of peripheral antigen to be detected [14]. Due to their speed and cost-effectiveness, antigen tests are widely employed worldwide. Serologic assays (complement fixation, immunodiffusion, and Western blot) are less reliable in disseminated disease due to the host’s immunosuppressed status. A meta-analysis reported an overall sensitivity of 58% for antibody detection across five studies on disseminated disease [15]. PCR methods, targeting various genomic regions of *H. capsulatum* (conventional, nested, real-time PCR), show promise with potentially superior sensitivity and specificity compared to serologic or antigen tests. However, their use is limited by the lack of FDA-approved, commercially available assays [16,17].

Treatment of disseminated histoplasmosis in immunocompromised individuals depends on disease severity [18]. Itraconazole (200 mg thrice a day for three days, followed by 200 mg twice a day) for at least 12 weeks is the recommended treatment in mild to moderate disease, including mild to moderate acute pulmonary histoplasmosis, chronic cavitary pulmonary histoplasmosis, pericarditis, mediastinal lymphadenitis, and mild to moderate progressive disseminated histoplasmosis. Severe disease warrants induction with liposomal Amphotericin B for one to two weeks, followed by itraconazole as above for a total of at least 12 months. Severe disease includes moderately severe to severe acute pulmonary histoplasmosis, characterized by acute respiratory distress syndrome or hypoxic respiratory failure, or moderately severe to severe progressive disseminated histoplasmosis, characterized by end-organ damage, including renal, hepatic impairment, or multi-organ failure [18]. CNS involvement requires liposomal amphotericin B for four to six weeks, followed by itraconazole (200 mg two to three times a day) for no less than one year and until CSF findings normalise, including *Histoplasma* antigen levels. Lifelong itraconazole suppression may be necessary in persistently immunosuppressed individuals or those with relapsing disease despite therapy [18]. Antigen levels should be followed during treatment and for up to 12 months after completion to monitor for relapse; however, low-level antigenuria alone does not necessarily signify treatment failure in the setting of clinical resolution. Fluconazole shows lower in vitro activity and clinical efficacy than Itraconazole and is linked to slower antigen clearance. Resistance to fluconazole may confer cross-resistance to voriconazole (41% of cases) but not to isavuconazole or posaconazole. Posaconazole, which has strong in vitro activity against *H. capsulatum*, outperformed Itraconazole in animal models and has shown success in salvage therapy [19]. Therapeutic drug monitoring (TDM) is recommended for patients receiving itraconazole, as approximately 20% of patients may require dose adjustments for sub- or supra-therapeutic drug levels. An itraconazole trough concentration between >1 mg/L and <3-4 mg/L, measured by chromatographic assay, is associated with therapeutic efficacy while minimizing the risk of toxicity [20].

In endemic areas where histoplasmosis incidence exceeds 10 cases per 100 patient-years, HIV-infected patients with CD4 counts below 150 cells/mm³ should be treated with 200 mg a day of Itraconazole for prophylaxis [18].

## Conclusions

Disseminated histoplasmosis occurs commonly in immunocompromised individuals, especially in patients with AIDS, due to poor cell-mediated immunity. Our report aligns with previous studies showing disseminated disease in these patients, most commonly involving lymph nodes and gastrointestinal tract, presenting with diarrhoea and abdominal pain, diagnosed on direct visualisation of the fungus in tissue specimens. Histoplasmosis is an important consideration in differential diagnoses in patients with AIDS, even if they are residing outside of endemic regions, who present with pulmonary or gastrointestinal symptoms, lymphadenopathy, meningitis, or fever of unknown origin, as this disease can be fatal if untreated.

## References

[1] Linder KA, Kauffman CA (2019). Histoplasmosis: epidemiology, diagnosis, and clinical manifestations. Curr Fungal Infect Rep.

[2] Hajjeh RA (1995). Disseminated histoplasmosis in persons infected with human immunodeficiency virus. Clin Infect Dis.

[3] Deepe GS Jr (1988). Protective immunity in murine histoplasmosis: functional comparison of adoptively transferred T-cell clones and splenic T cells. Infect Immun.

[4] Kennedy CC, Limper AH (2007). Redefining the clinical spectrum of chronic pulmonary histoplasmosis: a retrospective case series of 46 patients. Medicine (Baltimore).

[5] Goodwin R, Shapiro JL, Thurman GH, Thurman SS, Des-Prez RM (1980). Disseminated histoplasmosis: clinical and pathologic correlations. Medicine.

[6] Wheat LJ, Connolly-Stringfield PA, Baker RL (1990). Disseminated histoplasmosis in the acquired immune deficiency syndrome: clinical findings, diagnosis and treatment, and review of the literature. Medicine.

[7] Couppié P, Herceg K, Bourne-Watrin M (2019). The broad clinical spectrum of disseminated histoplasmosis in HIV-infected patients: a 30 years' experience in French Guiana. J Fungi (Basel).

[8] Wheat LJ, Musial CE, Jenny-Avital E (2005). Diagnosis and management of central nervous system histoplasmosis. Clin Infect Dis.

[9] Villareal K, Price A, Pasqualotto AC, Bahr NC (2023). The current and future states of diagnostic tests for histoplasmosis with a focus on people with HIV and disseminated histoplasmosis. J Fungi (Basel).

[10] Poplin V, Smith C, Milsap D, Zabel L, Bahr NC (2021). Diagnosis of pulmonary infections due to endemic fungi. Diagnostics (Basel).

[11] Wheat LJ, Azar MM, Bahr NC, Spec A, Relich RF, Hage C (2016). Histoplasmosis. Infect Dis Clin North Am.

[12] Pasqualotto AC, Queiroz-Telles F, Chebabo A (2023). The "Histoplasmosis Porto Alegre manifesto"-addressing disseminated histoplasmosis in AIDS. PLoS Negl Trop Dis.

[13] Yetmar ZA, Marty PK, Clement J, Miranda C, Wengenack NL, Beam E (2025). Executive summary of state-of-the-art review: modern approach to nocardiosis-diagnosis, management, and uncertainties. Clin Infect Dis.

[14] Hage CA, Azar MM, Bahr N, Loyd J, Wheat LJ (2015). Histoplasmosis: up-to-date evidence-based approach to diagnosis and management. Semin Respir Crit Care Med.

[15] Caceres DH, Knuth M, Derado G, Lindsley MD (2019). Diagnosis of progressive disseminated histoplasmosis in advanced HIV: a meta-analysis of assay analytical performance. J Fungi (Basel).

[16] Maubon D, Simon S, Aznar C (2007). Histoplasmosis diagnosis using a polymerase chain reaction method. Application on human samples in French Guiana, South America. Diagn Microbiol Infect Dis.

[17] Babady NE, Buckwalter SP, Hall L, Le Febre KM, Binnicker MJ, Wengenack NL (2011). Detection of Blastomyces dermatitidis and Histoplasma capsulatum from culture isolates and clinical specimens by use of real-time PCR. J Clin Microbiol.

[18] Wheat LJ, Freifeld AG, Kleiman MB, Baddley JW, McKinsey DS, Loyd JE, Kauffman CA (2007). Clinical practice guidelines for the management of patients with histoplasmosis: 2007 update by the Infectious Diseases Society of America. Clin Infect Dis.

[19] Wheat LJ, Connolly P, Smedema M (2006). Activity of newer triazoles against Histoplasma capsulatum from patients with AIDS who failed fluconazole. J Antimicrob Chemother.

[20] Arnold SR, Spec A, Baddley JW (2026). 2025 clinical practice guideline update by the Infectious Diseases Society of America on histoplasmosis: treatment of asymptomatic histoplasma pulmonary nodules (histoplasmomas) and mild or moderate acute pulmonary histoplasmosis in adults, children, and pregnant people. Clin Infect Dis.

